# Supplementary material for: The Microbial Mechanisms of a Novel Photosensitive Material (Treated Rape Pollen) in Anti-Biofilm Process under Marine Environment
Source: Int J Mol Sci. 2022 Mar 30;23(7):3837. doi: 10.3390/ijms23073837 (PMC8998240; doi:10.3390/ijms23073837)
Supplement: Supplementary file 1 [file ijms-23-03837-s001.zip › ijms-1625976-supplementary.pdf]

Supporting Information

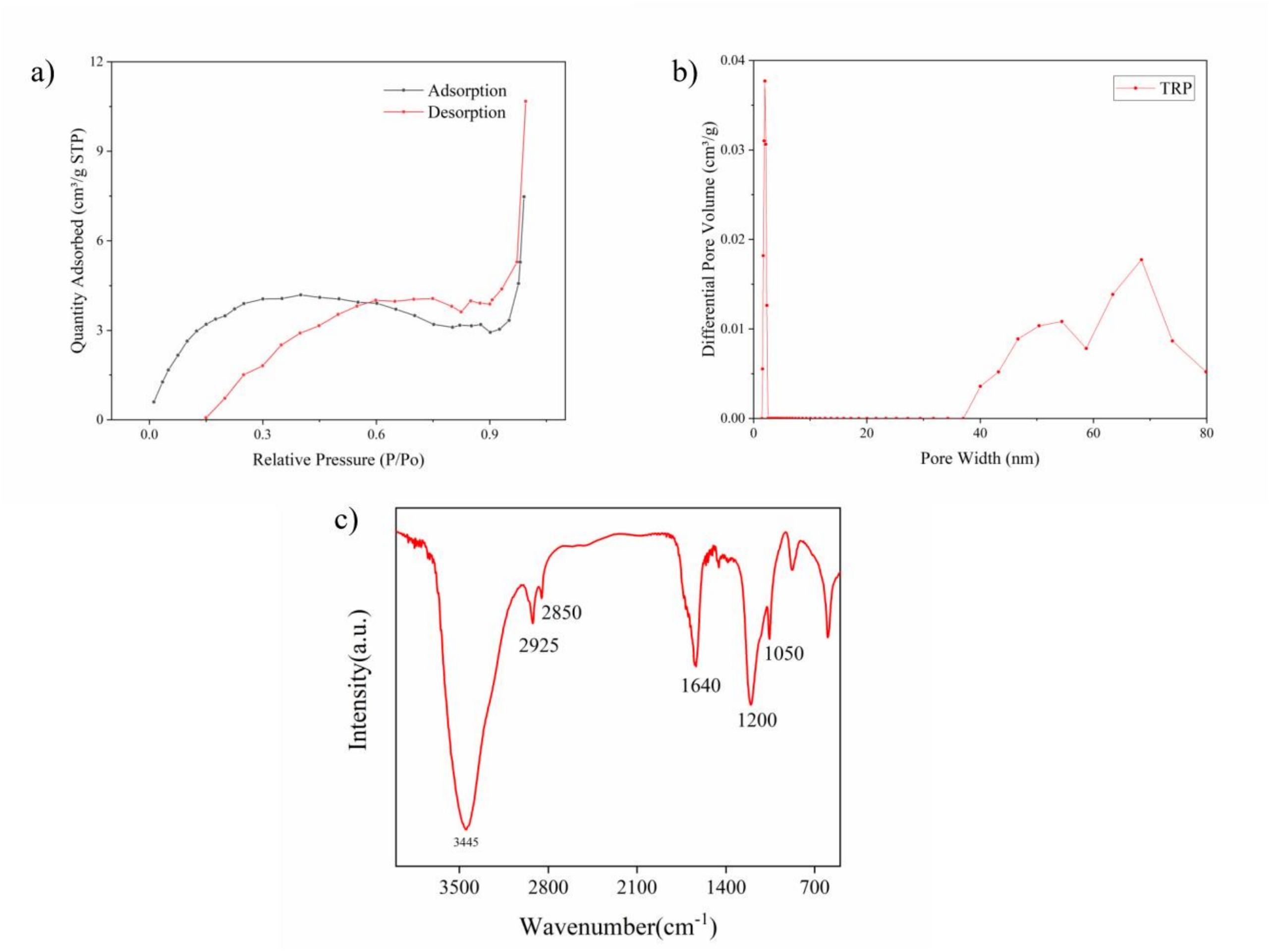

Figure S1. a) BET specific surface area, b) The Differential pore volume distribution and c) FTIR spectra of TRP.

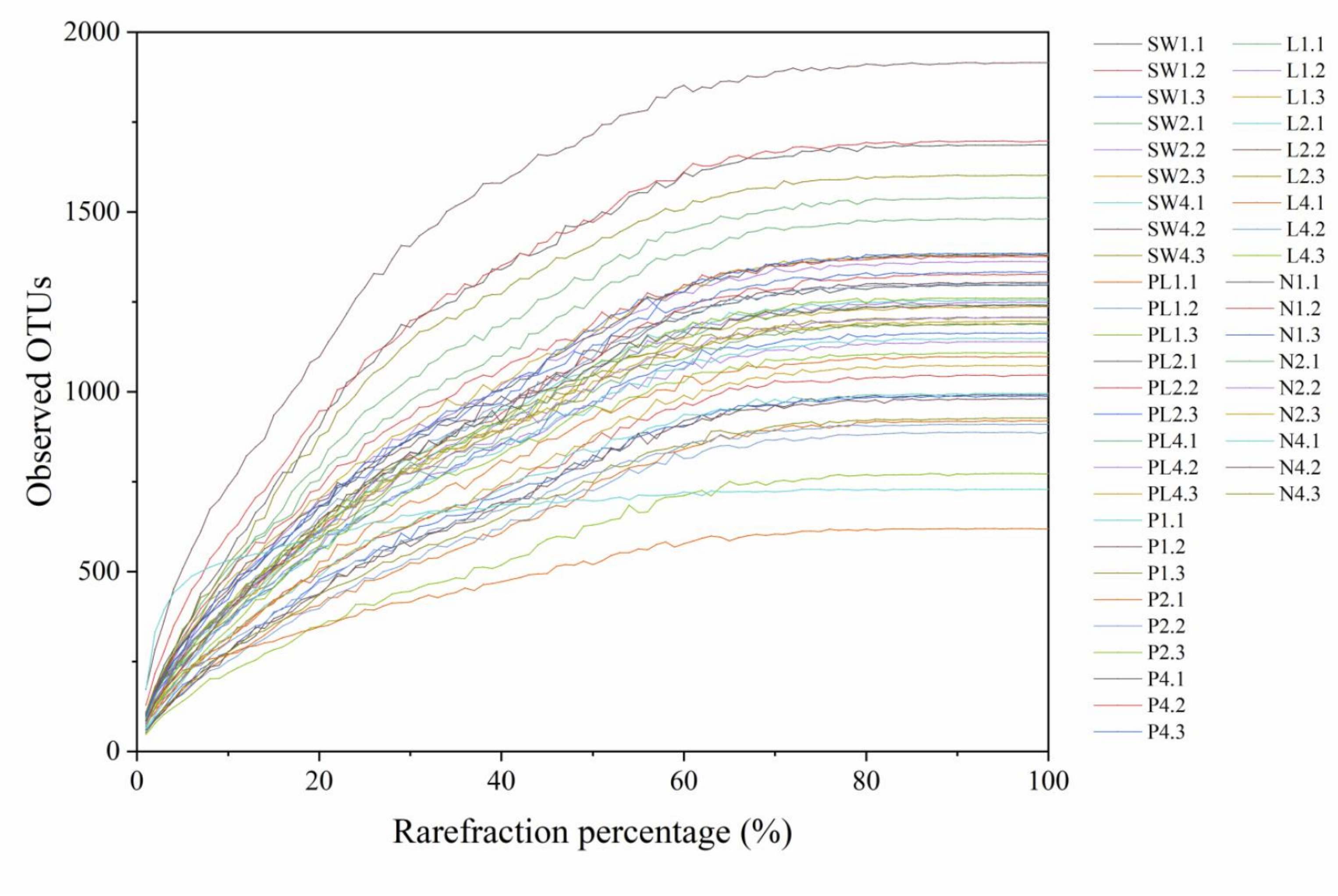

Figure S2. Rarefaction curves of observed OTUs and 16S rRNA gene sequence numbers.

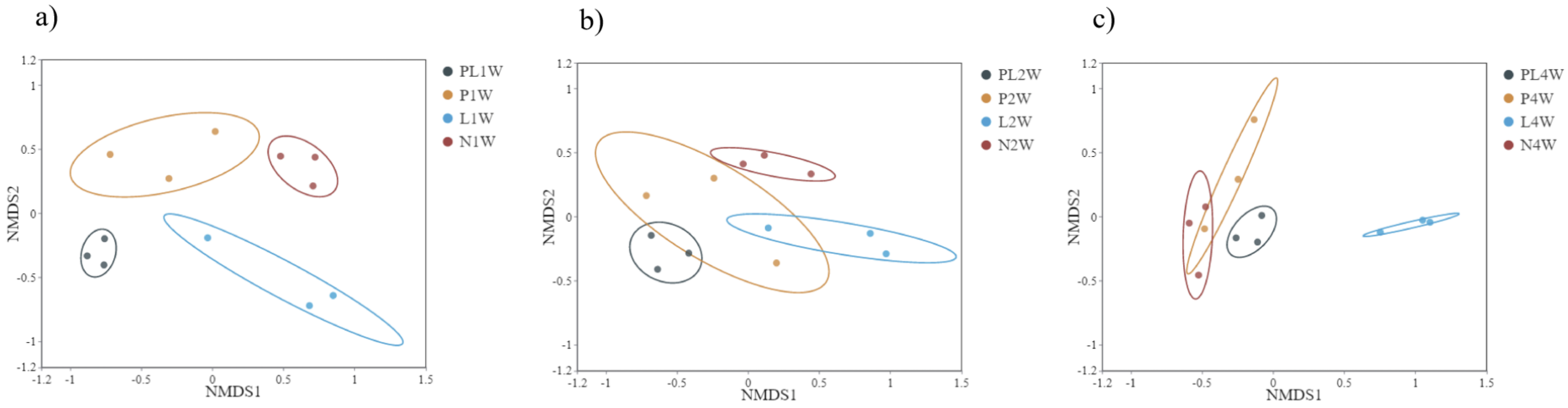

Figure S3. a) The Nonmetric Multidimensional Scaling (NMDS) among the various samples during different time points. a) 1 week, b) 2 weeks and c) 4 weeks.

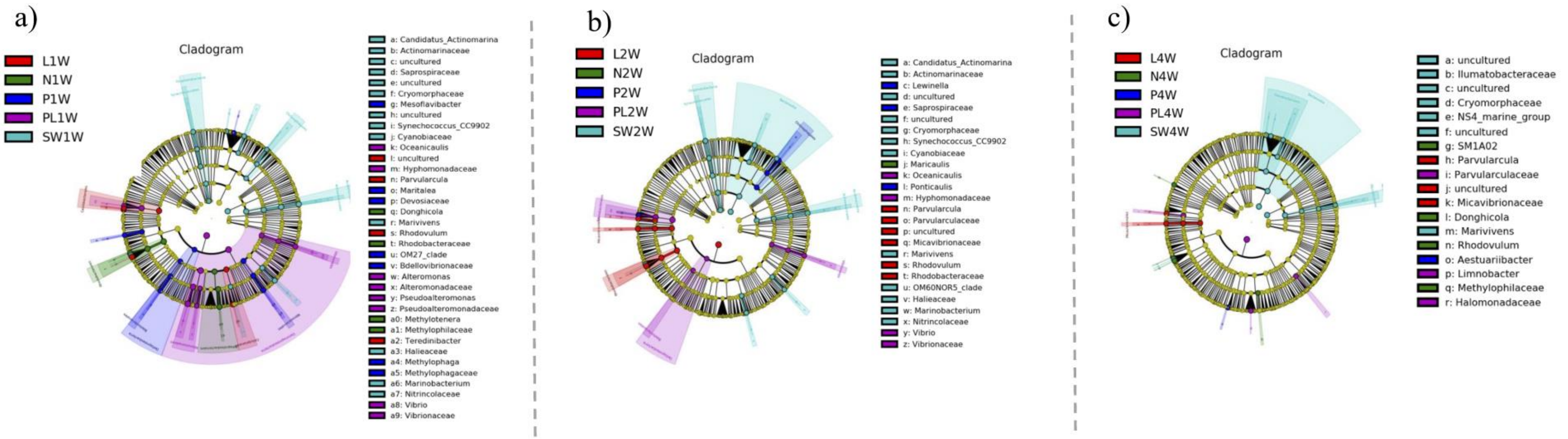

Figure S4. The Linear Discriminant Analysis Effect Size (LEfSe, LDA>4, from phylum to genus) of microbial communities structure on a) 1 week, b) 2 weeks and c) 4 weeks.

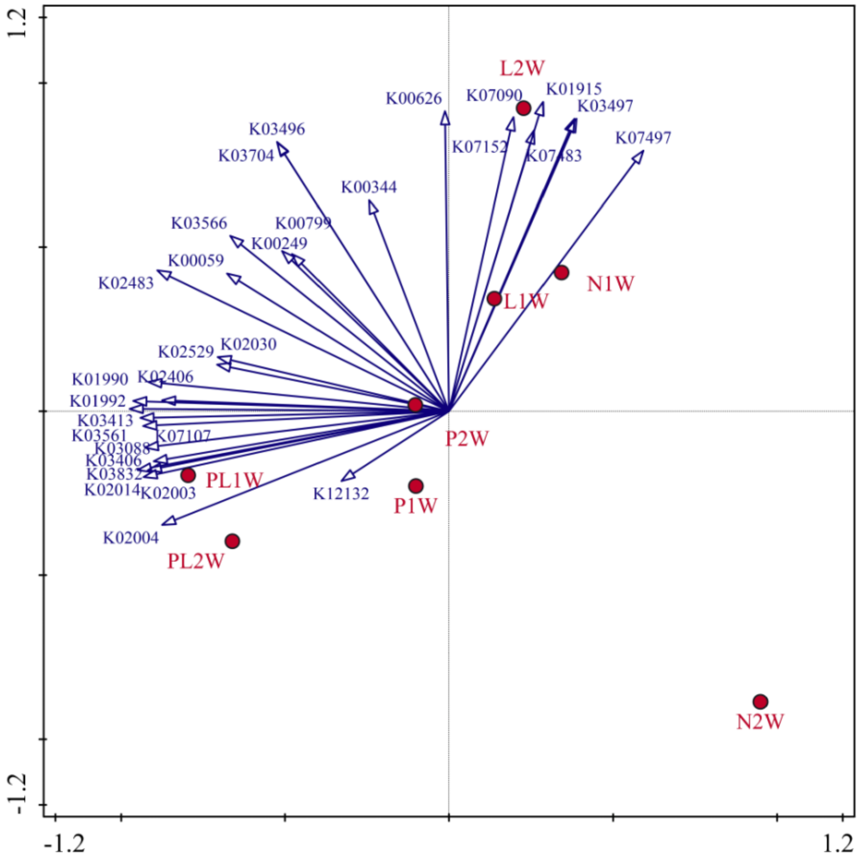

Figure S5. Redundancy Analysis (RDA) on the correlation between different biofilms and genes.

Table S1. Information of the 16S rRNA gene (V4-V5 region) amplicon sequences. Paired-end reads (read length 250 bp) sequenced using Illumina HiSeq 2500 platform were merged together for further analyses.

No. for analysis	Seawater/Biofilm 16S amplicons	Total sequences	No. of OTUs	Filter/ Biofilm substrata	Sampling time/ Durations for biofilm formation
SW1.1	Seawater	65618	1296	0.22 µm	1st week
SW1.2	Seawater	61807	1326	0.22 µm	1st week
SW1.3	Seawater	63621	1163	0.22 µm	1st week
SW2.1	Seawater	57751	1188	0.22 µm	2se weeks
SW2.2	Seawater	64621	1249	0.22 µm	2se weeks
SW2.3	Seawater	66208	1236	0.22 µm	2se weeks
SW4.1	Seawater	72154	1255	0.22 µm	4th weeks
SW4.2	Seawater	59369	1915	0.22 µm	4th weeks
SW4.3	Seawater	70550	1189	0.22 µm	4th weeks
PL1.1	TRP with light	71459	920	904L steel alloy	1st week
PL1.2	TRP with light	74243	888	904L steel alloy	1st week
PL1.3	TRP with light	61161	773	904L steel alloy	1st week
PL2.1	TRP with light	70201	991	904L steel alloy	2se weeks
PL2.2	TRP with light	69206	1046	904L steel alloy	2se weeks
PL2.3	TRP with light	57817	988	904L steel alloy	2se weeks
PL4.1	TRP with light	60189	1539	904L steel alloy	4th weeks
PL4.2	TRP with light	60218	1362	904L steel alloy	4th weeks
PL4.3	TRP with light	58073	1381	904L steel alloy	4th weeks
P1.1	TRP only	73422	995	904L steel alloy	1st week
P1.2	TRP only	68436	1304	904L steel alloy	1st week

P1.3	TRP only	62604	1206	904L steel alloy	1st week
P2.1	TRP only	71089	1097	904L steel alloy	2se weeks
P2.2	TRP only	63643	1300	904L steel alloy	2se weeks
P2.3	TRP only	56856	1260	904L steel alloy	2se weeks
P4.1	TRP only	67934	1686	904L steel alloy	4th weeks
P4.2	TRP only	60002	1697	904L steel alloy	4th weeks
P4.3	TRP only	66936	1333	904L steel alloy	4th weeks
L1.1	light only	72562	1481	904L steel alloy	1st week
L1.2	light only	69581	1139	904L steel alloy	1st week
L1.3	light only	62898	1073	904L steel alloy	1st week
L2.1	light only	69359	1148	904L steel alloy	2se weeks
L2.2	light only	71503	980	904L steel alloy	2se weeks
L2.3	light only	69620	927	904L steel alloy	2se weeks
L4.1	light only	79542	620	904L steel alloy	4th weeks
L4.2	light only	76719	910	904L steel alloy	4th weeks
L4.4	light only	62336	1108	904L steel alloy	4th weeks
N1.1	No TRP and no light	68541	1380	904L steel alloy	1st week
N1.2	No TRP and no light	74771	1376	904L steel alloy	1st week
N1.3	No TRP and no light	59722	1385	904L steel alloy	1st week
N2.1	No TRP and no light	68910	1242	904L steel alloy	2se weeks
N2.2	No TRP and no light	64573	1207	904L steel alloy	2se weeks
N2.3	No TRP and no light	58654	1196	904L steel alloy	2se weeks
N4.1	No TRP and no light	64277	729	904L steel alloy	4th weeks
N4.2	No TRP and no light	64421	1240	904L steel alloy	4th weeks
N4.3	No TRP and no light	54545	1602	904L steel alloy	4th weeks

Table S2. The sequence profile of the metagenomic paired-end reads (read length 150 bp) sequenced using Illumina HiSeq 2500 platform.

Metagenome	Raw reads	Clean reads	N50 of contigs	Total length of contigs (bp)	Total number of contigs	GC%
PL1W	79,366,220	69,062,308	1,434	285,459,196	228,040	51.66
PL2W	70,330,702	60,575,726	1,823	329,780,398	232,630	52.04
P1W	75,864,406	65,255,954	1,337	538,207,523	449,267	53.57
P2W	89,146,598	75,768,092	1,267	567,051,497	490,790	53.66
L1W	78,586,648	68,115,450	1,115	485,838,727	457,325	50.58
L2W	90,441,092	76,518,124	1,195	329,659,655	295,644	50.28
N1W	73,411,976	62,754,276	1,180	531,208,478	478,763	52.58
N2W	76,364,314	63,644,202	1,185	503,301,901	456,516	46.75

Table S3. Environmental physicochemical parameters of seawater at each sampling time.

Sampling Time	Temperature (℃)	Salinity (‰)	pH	Chlorophyll （μg/l）	TOC (mg/L)	NH_4^+-N (mg/L)	NO_2^-N (mg/L)	NO_3^-N (mg/L)	PO_4^{3-}P (mg/L)
0 week	28.55	32.66	8.65	0.18	2.03	0.189	0.007	0.035	0.027
1 Week	28.19	33.66	8.60	0.15	2.08	0.155	0.002	0.018	0.020
2 Week	27.63	33.78	8.70	0.20	1.93	0.216	0.010	0.060	0.029
4 Week	29.09	31.91	8.74	0.17	1.84	0.142	0.005	0.054	0.035

Table S4. Indicator taxa in each group (node degree≥5, betweenness centrality value ≤1000 and relative abundances >2.0% of the total number of bacterial communities).

Group	Indicator taxa
SW	*Marivivens, Synechococcus_CC9902, Marinobacterium*
PL	*Alteromonas, Parvularcula, Teredinibacter, Labrenzia, Aestuariibacter, Maricaulis*
P	*Alteromonas, Aestuariibacter, Teredinibacter, Donghicola, Methylophaga*
L	*Alteromonas, Rhodovulum, Parvularcula, Teredinibacter, Candidatus_Actinomarina*
N	*Donghicola, Methylotenera, Aestuariibacter*

Table S5. Permutational multivariate analysis of variance (W is the week).

Group	R^2	Pr(>F)
SW/PL/P/L/N(1W/2W/4W)	0.732	0.001
SW(1W/2W/4W)	0.239	0.524
PL(1W/2W/4W)	0.642	0.004
P(1W/2W/4W)	0.402	0.016
L(1W/2W/4W)	0.636	0.003
N(1W/2W/4W)	0.57	0.005
PL/P/L/N(1W)	0.686	0.001
PL/P/L/N(2W)	0.611	0.001
PL/P/L/N(4W)	0.583	0.003
